# Biological Evaluation of DNA Biomarkers in a Chemically Defined and Site-Specific Manner

**DOI:** 10.3390/toxics7020036

**Published:** 2019-06-25

**Authors:** Ke Bian, James C. Delaney, Xianhao Zhou, Deyu Li

**Affiliations:** 1 Department of Biomedical and Pharmaceutical Sciences, College of Pharmacy, University of Rhode Island, Kingston, RI 02881, USA; kebian@uri.edu (K.B.); xianhao_zhou@my.uri.edu (X.Z.); 2 Visterra, Inc., 275 Second Avenue, Waltham, MA 02451, USA; delaney@mit.edu

**Keywords:** DNA lesion, DNA damage, shuttle vector technique, replication block, mutagenicity, mutational spectrum, mutational signature, DNA repair, DNA adduct bypass, site-specific mutagenesis

## Abstract

As described elsewhere in this Special Issue on biomarkers, much progress has been made in the detection of modified DNA within organisms at endogenous and exogenous levels of exposure to chemical species, including putative carcinogens and chemotherapeutic agents. Advances in the detection of damaged or unnatural bases have been able to provide correlations to support or refute hypotheses between the level of exposure to oxidative, alkylative, and other stresses, and the resulting DNA damage (lesion formation). However, such stresses can form a plethora of modified nucleobases, and it is therefore difficult to determine the individual contribution of a particular modification to alter a cell’s genetic fate, as measured in the form of toxicity by stalled replication past the damage, by subsequent mutation, and by lesion repair. Chemical incorporation of a modification at a specific site within a vector (site-specific mutagenesis) has been a useful tool to deconvolute what types of damage quantified in biologically relevant systems may lead to toxicity and/or mutagenicity, thereby allowing researchers to focus on the most relevant biomarkers that may impact human health. Here, we will review a sampling of the DNA modifications that have been studied by shuttle vector techniques.

## 1. Introduction

**Figure 1:**
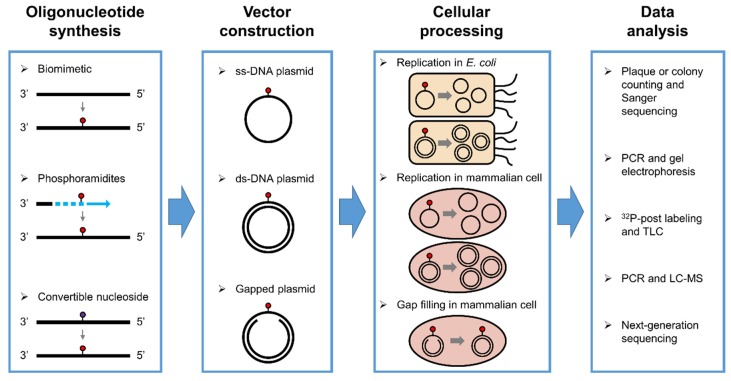
Schematic overview of the shuttle vector-based methods for evaluating DNA biomarkers.

The human genome is constantly exposed to and damaged by endogenous chemicals, such as reactive oxygen species, lipid peroxidation intermediates, and alkylating agents. These electrophilic reactive chemicals, as well as environmental carcinogens and administered drugs, are known to generate various DNA adducts [1,2,3]. Some of the adducts block DNA replication or cause mutations and have been used as biomarkers to monitor the level of DNA damage or of disease progression [4,5,6]. One of the major goals for researchers is to understand the deleterious consequences of those lesions within the cell or animal. Among the different methods for studying the biological effects of the adducts, use of shuttle vectors containing a chemically defined lesion at a specific site has provided information about the biological and toxicological properties of the adduct [4,7]. The shuttle vector-based methods normally involve the steps outlined in Figure 1. *Oligonucleotide synthesis*: An oligonucleotide (oligo) containing a structurally defined lesion at a specific site is made either through a biomimetic route (in situ formation by direct chemical reaction, followed by HPLC purification of site-specifically modified oligo), or purely synthetically using a normal or convertible nucleoside phosphoramidite, etc. *Vector construction*: An ss- or ds-DNA vector containing the modified oligo is built by cutting the parent vector with one or a pair of restriction endonuclease(s), followed by ligation of the 5′-phosphorylated modified oligo. *Cellular processing*: The vector is transfected into different types of cells (e.g., *Escherichia. coli* (*E. coli*) or mammalian), and cellular polymerases are allowed to replicate or transcriptionally bypass the lesion under different repair or bypass conditions. *Data analysis*: DNA is extracted, amplified using PCR, and the biological outcomes are analyzed, which include the ability of the lesion/adduct to block polymerases or cause a mutation when processed by a polymerase during cellular replication. This assessment could be done by plaque or colony counting and picking with Sanger sequencing, ^32^P-post labeling and thin-layer chromatography (TLC), liquid chromatography-mass spectrometry (LC-MS), next-generation sequencing (NGS), etc. [4,5,7,8,9]. The shuttle vector-based method was initially introduced by Essigmann [7,9,10,11], further developed and utilized by Wang [4,5], Moriya [12,13], Livneh [14,15], Greenberg [16,17], Basu [18,19], Lloyd [20,21], Loechler [22,23], Fuchs [24,25], Pagès [26,27], and others. Several informative review articles have been written by these authors on designing and applying the methods.

In this work, we will review a variety of DNA biomarkers or probes that have been studied using the shuttle vector techniques and briefly summarize their biological outcomes. In all cases, focus is placed on the effect of the lesion to block replication and to cause mutations. For the details regarding the formation of DNA damage and other properties of the lesions, please refer to the original literature or review articles. We apologize in advance to researchers whose work we could not include in this review. After detailed discussions on individual lesions, we will provide some perspectives on possible future directions.

## 2. Discussions on Individual Modifications

**Figure 2:**
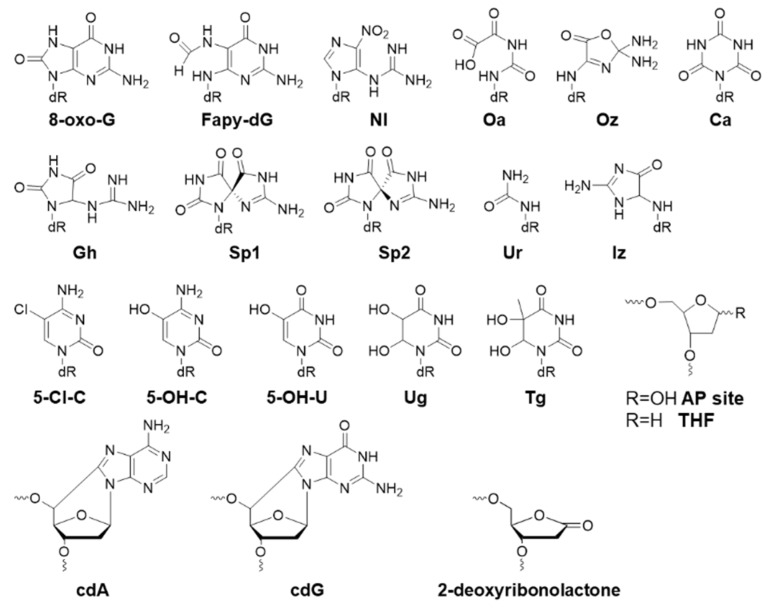
Structures of oxidative lesions.

**Figure 3:**
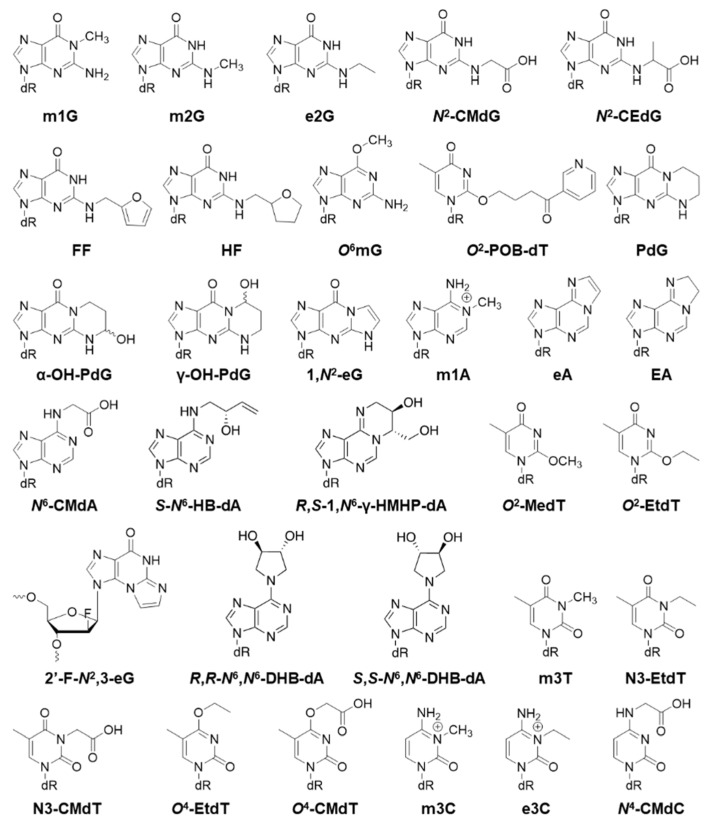

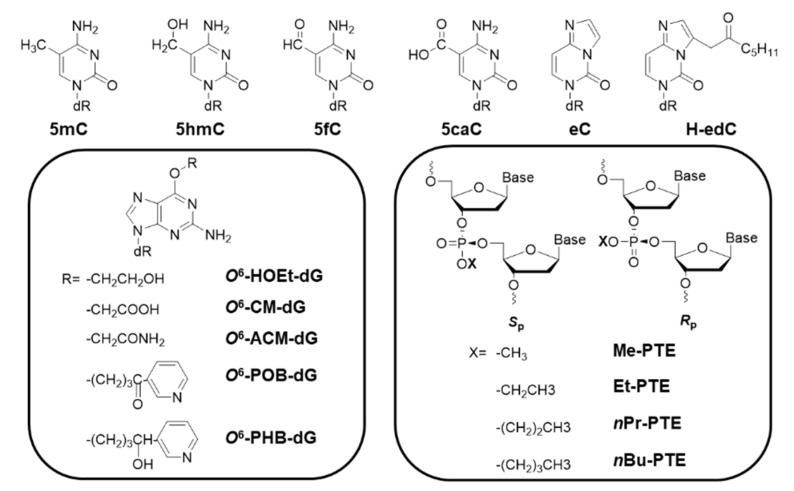
Structures of alkyl modifications.

**Figure 4:**
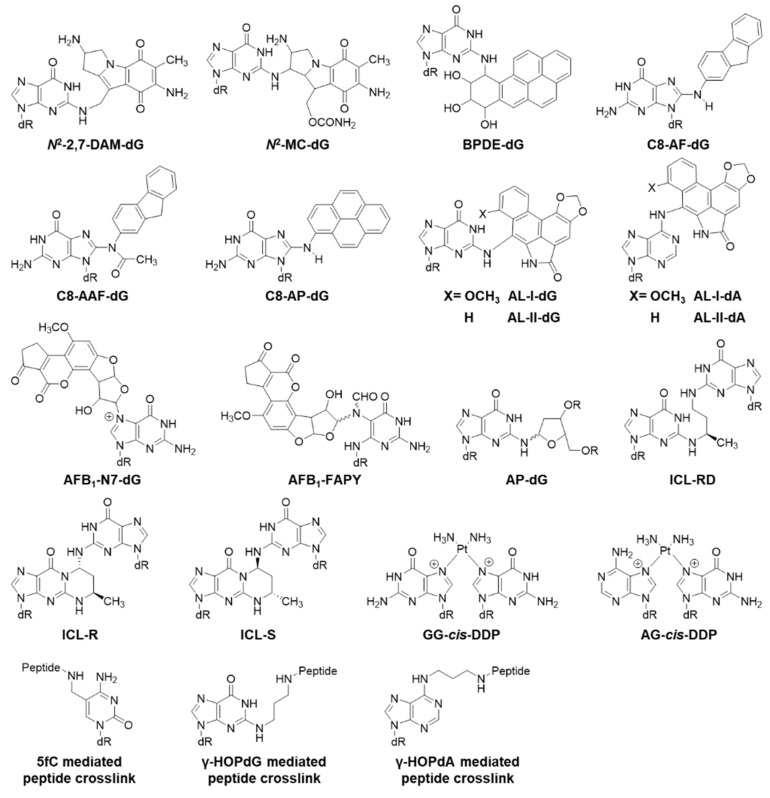
Structures of bulky and crosslinked lesions.

**Figure 5:**
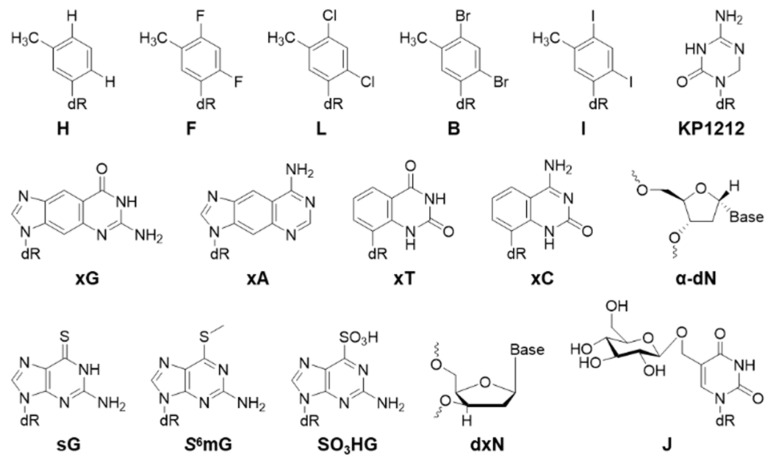
Structures of other nucleotide analogs.

Below, we will cover modified DNA structures generated from oxidative stress, alkylation, and other processes (Figure 2, Figure 3, Figure 4 and Figure 5). In the following sections, the biological effects of a certain lesion are briefly summarized. Please see Figure 2, Figure 3, Figure 4 and Figure 5 for chemical structures and Table 1 for detailed information.

### 2.1. Oxidative Biomarkers

All the structures of modifications covered in this section are displayed in Figure 2. 8-Oxo-7,8-dihydro-2′-deoxyguanosine (8-oxo-G) is not a strong block to replication, demonstrating greater than 80% bypass efficiency in *E. coli* [28]. Its mutagenic pairing with A during replication in wild type (WT) cells leads to a low amount of G>T mutation (3%) [28]. However, in MutY-cells (MutY: adenine glycosylase in 8-oxo-G:A base excision repair), the G>T mutation increases to 44% [29]. 8-oxo-G causes mainly G>T mutation with a frequency of 8% in human cells [28,29]. Thymidine glycol (Tg) is not a replication block, and it is not mutagenic in *E. coli*; however, tandem lesions of 8-oxoG and Tg are twice as effective as a single 8-oxo-G in blocking DNA replication, and the dual lesion is more mutagenic than 8-oxo-G [38]. Fapy-dG (N-(2-deoxy-α,β-d-erythropentofuranosyl)-N-(2,6-diamino-4-hydroxy-5-formamidopyrimidine)) strongly blocks replication by 60–70% in *E. coli*, but it is not very mutagenic, providing less than 2% G>T mutation [31]. Fapy-dG causes 10% G>T mutation in human cells [30]. 5-Guanidino-4-nitroimidazole (NI) strongly blocks replication (93%) in *E. coli*, giving mainly G>T (22%) and G>A (19%) mutations, and some G>C (9%) mutation as well [32]. Oxaluric acid (Oa) is toxic, blocking replication by 50%, causing nearly 100% G>T mutation in *E. coli* [28,31,34]. Oxazalone (Oz) strongly blocks replication and is very mutagenic, causing 86% G>T mutation [28]. Cyanuric acid lesion (Ca) blocks 35% replication in *E. coli*, and is very mutagenic with 95% G>T mutation [28]. Guanidinohydantoin (Gh) slightly blocks replication (25%), and it is highly mutagenic yielding 97% G>C and 2% G>T mutation [34]. Two stable stereoisomers of spiroiminodihydantoin (Sp1 and Sp2) are strong replication blocks (91%), and are both very mutagenic, causing mainly G>C (72% for Sp1 and 57% for Sp2) and G>T (27% for Sp1 and 41% for Sp2) mutations [34]. Urea lesion (Ur) is a strong replication block (90%) causing 54% G>T, 35% G>C, and 9% G>A mutations [29,35]. Imidazolone adduct (Iz) can be bypassed in *E. coli* with a 40% blockage in replication, essentially causing G>C (88%) mutation, with some G>A (2%) and G>T (1%) mutations [32]. 8,5′-Cyclo-2′-deoxyguanosine (cdG) is a strong replication block (89%) in *E. coli*, and knocking out pol V increases its replication block; it is mutagenic and causes 20% G>A mutation [36]. The 5′ S-diastereomer of cyclo-dG (S-cdG) also strongly blocks DNA replication (96%) in human cells, giving primarily G>T (35%) and G>A (20%) mutations [37]. 8,5′-Cyclo-2′-deoxyadenosine (cdA) is 31% bypassed in *E. coli*, but the bypass efficiency drops to 13% when pol V is removed from the cell [36]. It is mutagenic and causes A>T (11%) mutation [36]. The 5′ S-diastereomer of cyclo-dA (S-cdA) strongly blocks replication in human cells by 94% [37]. Knocking down pol η by siRNA decreases the bypass efficiency and mutagenicity of S-cdA [37]. 5-Chlorocytosine (5-Cl-dC) blocks replication (25%), forming a low level of C>T mutation (5%) in *E. coli* [39]. 5-Hydroxycytosine (5-OH-dC) is not mutagenic in *E. coli* [40]. 5-Hydroxyuracil (5-OH-dU, derived from 5-OH-dC) is very mutagenic providing 83% C>T mutation in *E. coli* [40]. 5,6-Dihydroxy-5,6-dihydrouracil (Ug) is also very mutagenic (80% C>T) in *E. coli* [40].

Tetrahydrofuran (THF) is a stable structural analog to the abasic site (AP site), which is not stable and may lead to further damages to the DNA strand. THF strongly blocks replication (>95%) and causes G>T (50%), G>C (26%), and G>A (7%) mutations; additionally, it causes 13% −1 frame shift mutation [28,29,32,34,80].

### 2.2. Alkyl Biomarkers

All the structures of modifications covered in this section are displayed in Figure 3. 1-Methyldeoxyguanosine (m1G) is a strong replication block either with or without the repair enzyme AlkB (85% and 97%); it mainly causes ~3% G>T mutation in WT *E. coli*, which increases to more than 50% in AlkB- *E. coli* (AlkB: alkyl DNA adduct direct reversal of damage repair protein) [9,33]. *N*^2^-methylguanine (m2G) weakly blocks replication by 10% in *E. coli*, there is no significant change when knocking out either AlkB or DinB (DinB: DNA polymerase IV), and a small amount of G>A mutation (3%) is seen [42]. *N*^2^-ethylguanine (e2G) does not block replication in *E. coli* and causes a low amount of G>A mutation (2%); eliminating AlkB and DinB does not change the replication bypass and mutagenicity significantly [42]. *N*^2^-carboxymethyl-2′-deoxyguanosine (*N*^2^-CMdG) and *N*^2^-(1-carboxyethyl)-2′-deoxyguanosine (*N*^2^-CEdG) do not block DNA replication and are not mutagenic in WT mammalian cells; however, each of them causes G>A (23%) and G>T (15%) mutations in mouse embryonic fibroblast (MEF) cells that are deficient in pol κ [43]. *N*^2^-CEdG blocks replication in *E. coli* [44]. The *R*-*N*^2^-CEdG is a stronger replication block (61%) than *S*-*N*^2^-CEdG (25%); however, neither of them are mutagenic [44]. *N*^2^-furfurylguanine (*N*^2^-FF-dG) does not block replication in WT *E. coli*; however, it blocks replication about 72% in DinB- cells [42]. It is not very mutagenic with or without DinB [42]. 2-Tetrahydrofuran-2-yl-methylguanine (*N*^2^-HF-dG) is similar in structure to *N*^2^-FF-dG and strongly blocks replication (72%) only when DinB is knocked out, and causes only 2% G>C mutation [42]. *O*^6^-methylguanine (*O*^6^mG) is very mutagenic and leads to almost 100% G>A mutation in Ada/Ogt/UvrB triple knockout *E. coli* (Ada/Ogt: alkyl DNA adduct direct reversal of damage repair protein; UvrB: nucleotide excision repair) [45,46]. *N*-Nitroso compounds induce DNA lesions: *O*^6^-pyridyloxobutyl-dG (*O*^6^-POB-dG), *O*^6^-pyridylhydroxybutyl-dG (*O*^6^-PHB-dG), *O*^6^-carboxymethyl-dG (*O*^6^-CMdG), which have two structural analogs: *O*^6^-aminocarbonylmethyl-dG (*O*^6^-ACM-dG) and *O*^6^-hydroxyethyl-dG (*O*^6^-HOEt-dG) [47]. *O*^6^-POB-dG slightly blocks DNA replication and induces G>A (90%) transition and G>T (2.5%) transversion in *E. coli* [47]. *O*^6^-PHB-dG is a moderate impediment to DNA replication and causes G>A (95%) mutation exclusively in *E. coli* [47]. *O*^6^-CMdG strongly inhibits replication in *E. coli*, but causes moderate G>A (10%) mutation [47]. *O*^6^-ACM-dG and *O*^6^-HOEt-dG are two analogs of *O*^6^-CM-dG. Both *O*^6^-ACM-dG (2% bypass) and *O*^6^-HOEt-dG (15% bypass) strongly block DNA replication [47]. They also induce G>A mutation with 30% and 40% frequencies, respectively [47]. Major acrolein-dG adducts include 8α and 8β isomers of 3H-8-hydroxy-3-(β-D-2′-deoxyribofuranosyl)-5,6,7,8-tetrahydropyrido[3,2-a]purine-9-one (γ-OH-PdG), 6α and 6β isomers (α-OH-PdG), and 1,*N*^2^-(1,3-propano)-2′-deoxyguanosine (PdG) [12]. The bypass efficiency for γ-OH-PdG is 73% compared to dG control in human cells, and γ-OH-PdG is not very mutagenic (<1%) [12]. α-OH-PdG strongly blocks DNA replication with a bypass efficiency of 17% in human cells and it causes G>T (11%) mutation [13]. PdG strongly blocks replication in human cells and mainly causes 6% G>T mutation [12]. Most of the derivatives of PdG moderately block DNA replication in human cells and cause mainly G>T mutation (2–8%) [81]. 1,*N*^2^-ethenoguanine (1,*N*^2^-eG) is a strong replication blocker (96%) in *E. coli* and causes G>A and G>T mutation by 6% for both, plus a small amount of G>C (2%) mutation; it also causes −1 and −2 frame shift mutations (5%), and knocking out AlkB leads to higher replication block and almost doubles the mutagenicity [8]. 2′-Fluoro-*N*^2^,3-*ε*-2′-deoxyarabinoguanosine (2′-F-*N*^2^,3-eG), a stable analog of *N*^2^,3-ethenoguanine (*N*^2^,3-eG), blocks replication by 79%, and causes 30% G>A mutation in *E. coli*, with AlkB having no significant influence in its replication bypass and mutagenicity [8].

1-Methyldeoxyadenosine (m1A) strongly blocks replication in AlkB- *E. coli* (88%), but it is not very mutagenic, causing <1% A>T mutation; m1A does not block replication in AlkB+ *E. coli* cells [9]. 1,*N*^6^-ethenoadenine (eA) weakly blocks replication by 4% in WT *E. coli*, but significantly blocks replication (95%) when AlkB is knocked out; likewise, eA is not mutagenic in WT *E. coli*, but shows strong mutagenicity in AlkB- cells (25% A>T mutation) [33,49]. Bypass efficiency of eA in human cells is 17% [50]. 1,*N*^6^-ethanoadenine (EA) does not block replication in WT *E. coli*, but strongly blocks replication by 86% when AlkB is removed; it is not very mutagenic in either WT or AlkB- cells, causing only 2% A>C mutations [49]. *N*^6^-carboxymethyl-2′-deoxyadenosine (*N*^6^-CMdA) minimally blocks replication in *E. coli* and is not mutagenic [36]. *S*-*N*^6^-HB-dA (HB = 2-hydroxy-3-buten-1-yl) and *R*,*R*-*N*^6^,*N*^6^-DHB-dA (DHB = 2,3-dihydroxybutan-1,4-diyl) do not block DNA replication and are not mutagenic in *E. coli* [51]. *S*,*S*-*N*^6^,*N*^6^-DHB-dA moderately inhibits replication with a 60% bypass efficiency, and causes minimal 1% A>G mutation [51]. *R*,*S*-1,*N*^6^-*γ*-HMHP-dA (HMHP = 2-hydroxy-3-hydroxymethylpropan-1,3-diyl) strongly inhibits DNA replication but causes only 2% A>T mutation [51].

*O*^2^-Methylthymidine (*O*^2^-Me-dT) can be bypassed by 55% in human cells and mainly causes T>A mutation (56%) [53]. *O*^2^-[4-(3-pyridyl-4-oxobut-1-yl]thymidine (*O*^2^-POB-dT) exhibits genotoxicity showing 26% bypass efficiency and is mutagenic with 47% T>A transversion [53]. Both *O*^2^-Me-dT and *O*^2^-POB-dT strongly block DNA replication in *E. coli* (95% and 97%) [54]. *O*^2^-Me-dT induces 10% T>A and 10% T>G mutations [54]. *O*^2^-POB-dT induces 38% T>G and 12% T>A mutations [54]. *O*^2^-Ethylthymidine (*O*^2^-EtdT) is a strong replication block (79%) in *E. coli*, and knocking out pol IV increases the blocking activity, while knocking out pol V increases the replication block even more [55]. It is very mutagenic and forms T>C (35%), T>A (15%), and T>G (5%) mutations, and mutation frequency drops when pol V is knocked out [55]. The bypass efficiency of *O*^2^-dT alkyl adducts in *E. coli* depends on the size of the alkyl lesion [82]. More than 20% of adducts can be bypassed during replication for ethyl and methyl substitutions, but less than 10% can be bypassed for propyl, and less than 5% for butyl adducts, with the major mutation type being T>C point mutation [82]. *O*^2^-alkyldT lesions strongly inhibit DNA replication (40–85%) in mammalian cells [52]. The blockage effect increases with the size and branching of the alkyl groups [52]. These lesions cause T>A and T>G mutations [52]. 3-Methyldeoxythymidine (m3T) strongly blocks replication in *E. coli* by 94% and is very mutagenic, generating mainly T>A (32%) transversion mutation; eliminating AlkB slightly increases its replication blocking power and mutagenicity [9]. N3-Ethylthymidine (N3-EtdT) strongly blocks replication by 83% in *E. coli*, and knocking out pol V or pol IV increases its blocking activity; it is very mutagenic causing T>A (21%), T>C (15%) and T>G (3%) mutations, and removing pol V eliminates the mutagenicity of this adduct [55]. N3-carboxymethylthymidine (N3-CMdT) strongly blocks replication by 45% in *E. coli*, with the major mutation being T>A (66%); and knocking out pol V slightly increases the mutation rate; however, knocking out pol IV decreases the mutation rate [36]. *O*^4^-carboxymethylthymidine (*O*^4^-CMdT) is a strong replication block (51%) and very mutagenic, causing 86% T>C mutation [36]. *N*^3^-CMdT, *O*^4^-CMdT and *O*^6^-carboxymethyl-dG (*O*^6^-CMdG) moderately block DNA replication in human cells [48]. *N*^3^-CMdT causes T>A (81%) mutation; *O*^4^-CMdT causes T>C (68%) mutation; *O*^6^-CMdG causes G>A (6.4%) mutation; neither *N*^6^-CMdA nor *N*^4^-CMdC block replication or induce mutation [48]. *O*^4^-Ethylthymidine (*O*^4^-EtdT) does not strongly block replication (24%) in WT *E. coli*, but it cannot be efficiently bypassed in pol II/IV/V triple knock out cells [55]. The major mutation of *O*^4^-EtdT is T>C (84%) transition; however, it does not cause mutations in *E. coli* lacking pol V [55]. *O*^4^-Alkylthymidine (*O*^4^-alkyldT) lesions moderately block DNA replication in human cells; pol ι and pol ζ promote the bypass of all *O*^4^-alkyldT lesions except *O*^4^-MedT [56]. The *O*^4^-alkyldT lesions induce only T>C transition mutations in cells [56].

3-Methyldeoxycytidine (m3C) has been demonstrated to strongly block replication (>90%) and generate mainly C to T (50%) and C to A mutations (30%) in the AlkB- *E. coli* cell [9]. However, the lesion is not mutagenic and not blocked by the replicative polymerases in the WT (AlkB+) cell [9]. 3-Ethyldeoxycytidine (e3C) does not block replication in *E. coli*; however, it dramatically blocks replication when knocking out AlkB (91%) [9]. e3C causes 17% C>T, 11% C>A, and 2% C>G mutations in AlkB- *E. coli*, but is not mutagenic in WT cells [9]. The m3C, e3C, and m1A lesions presumably have their methyl or ethyl groups removed by AlkB’s direct reversal of DNA alkyl damage mechanism prior to encountering the DNA polymerase [9]. *N*^4^-carboxymethyl-2′-deoxycytidine (*N*^4^-CMdC) weakly blocks replication (17%) and is not mutagenic in *E. coli* [36]. 5-Methylcytosine (5mC) and its derivatives 5-hydroxymethylcytosine (5hmC), 5-formylcytosine (5fC) and 5-carboxylcytosine (5caC) neither block replication nor cause mutation in *E. coli* [39,59]. 5mC also does not block replication in human cells, but there are some blockades of 5hmC (5%), 5fC (25%), and 5caC (28%) towards DNA replication in human cells [58]. 3,*N*^4^-ethenocytosine (eC) is a toxic adduct, which strongly blocks replication (76%) and leads to mutation with a pattern of dominant C>A (24%) and less C>T (11%) mutations in WT *E. coli*; in AlkB- cells, the blockage of replication increases to 87% and mutagenicity rises up to 49% C>A and 31% C>T mutations [33]. Lipid peroxidation-derived product 4-oxo-2(*E*)-nonenal reacts with dG, dA, and dC in DNA to form heptanone (H)-etheno (e) adducts [50]. H-edC shows strong DNA replication blocking in both *E. coli* (99%) and human cells (90%) [50]. It causes mainly C>G (40%) mutation in *E. coli*; however, mostly C>A (60%) and C>T (32%) mutations are seen in human cells [50].

5-Hydroxymethyluracil (5hmU) blocks replication by 20%, but it is not mutagenic in human cells [60]. The *S*_p_ alkyl phosphotriester (*S*_p_-alkyl-PTE) lesions display comparable replication bypass efficiency to unmodified DNA in *E. coli*; *S*_p_-Me-PTE is mutagenic causing TT>GT (50%) and TT>GC (15%) mutations [61]. In contrast, *R*_p_-alkyl-PTEs block DNA replication (30–70%) but are not mutagenic [61]. Interestingly, *n*Pr- and *n*Bu-PTEs exhibit higher bypass efficiencies than Me- and Et-PTEs [61].

### 2.3. Bulky Lesions

All the structures of modifications covered in this section are displayed in Figure 4. *N*-(deoxyguanosin-8-yl)-1-aminopyrene (C8-AP-dG) moderately blocks DNA replication in human cells [66]. *N*-acetyl-2-aminofluorene (C8-AAF-dG) strongly blocks replication [66]. 2-Aminofluorene (C8-AF-dG) slightly blocks replication [66]. All three adducts can be nearly bypassed in error free manner [66]. Aristolochic acids I and II (AA-I, AA-II) are found in all *Aristolochia* species and generate the aristolactam (AL) metabolite for forming DNA adducts with dA and dG. Both AL-II-dA and AL-II-dG strongly block DNA replication in MEF cells [63]. AL-II-dA causes 22% A>T mutation and AL-II-dG causes 9% G>T transversion [63]. Knocking out the rev3L gene dramatically suppresses bypass of AL-I-dA in MEF cells and abolishes A>T transversion [67]. Benzo[*a*]pyrene (BP)-7,8-diol-9,10-epoxide-*N*^2^-deoxyguanosine (BPDE-dG) is an adduct formed by benzo[*a*]pyrene; it predominantly miscodes with G>T (73%) and G>A (12%) mutations in WT MEF cells [68]. Knocking out rev1 gene decreases the bypass efficiency of BPDE-dG to 40% and changes the mutation frequency to 32% G>T and 18% G>A [68]. Knocking out the rev3L gene significantly decreases the bypass efficiency to 13% and decreases the mutation to 6% G>T [68]. Mitomycin C (MC) generates dG-N2-MC and dG-N2-2,7-Diaminomitosene (DAM) adducts, which can be bypassed 38% and 27% in human cells, respectively [62]. The major type of mutation is G>T mutation (18% for dG-N2-MC and 10% for dG-N2-2,7-DAM) [62]. Aflatoxin B_1_-N7-dG adduct (AFB_1_-N7-dG) is weakly mutagenic in *E. coli*, causing 1.5% G>T mutation [64]; and its FAPY adduct causes 14% G>T mutation [65].

### 2.4. Crosslinked Lesions

All the structures of modifications covered in this section are displayed in Figure 4. *N*^2^-guanine -*N*^2^-guanine interstrand crosslinks (ICLs), 3-(2-deoxyribos-1-yl)-5,6,7,8-(*N*^2^-deoxyguanosyl)-6(either R or S)-methylpyrimido[1,2-R]purine-10(3H)-one is a product induced by acetaldehyde/crotonaldehyde [69]. ICL-S and ICL-R moderately inhibit DNA replication in WT *E. coli*; however, their replication blocking effects increase in uvr- *E. coli* cells [69]. ICL-Rd is a moderate block in WT *E. coli*, but it almost completely blocks replication in uvr- cells [69]. All three lesions are weakly mutagenic in *E. coli* causing exclusively 5′-G>T (3%) transversions; no mutation is observed at the 3′-G site [69]. Similar mutations generated by these lesions are seen in human cells, except ICL-S has a slightly higher mutation frequency (6%) [69]. The crosslinks formed by *cis*-diaminedichloroplatinum (II) (*cis*-DDP, cisplatin) between two guanines or adenine-guanine strongly block DNA replication in *E. coli*, but they are not very mutagenic [72]. 5-Formylcytosine mediated peptide crosslink causes 7% C>T and 1% C>G mutation and 2% C deletion [73]. γ-Hydroxypropanodeoxyguanosine (γ-HOPdG) mediated crosslink between peptide and guanine is mutagenic, causing 5% G>T and 3% G>C mutations; however, the crosslink between peptide and γ-hydroxypropanodeoxyadenine (γ-HOPdA) is not mutagenic [20].

### 2.5. Other Nucleotide Analogs

All the structures of modifications covered in this section are displayed in Figure 5. A series of unnatural analogs of thymine (T) was developed by the Kool group to probe the biological requirements for DNA polymerases [74]. 3-Toluene-1-β-D-deoxyriboside (H) strongly blocks replication (95%) and is very mutagenic causing T>A (41%), T>C (5%), and T>G (4%) point mutations and −1 frame shift mutation (13%). 2,4-Difluoro-5-toluene-1-β-D-deoxyriboside (F) strongly blocks replication (87%) and is mutagenic causing T>A (9%), T>C (1%), and T>G (1%) mutations. 2,4-Dichloro-5-toluene-1-β-D-deoxyriboside (L) strongly blocks replication (80%) and is slightly mutagenic causing T>A (5%) mutation. 2,4-Dibromo-5-toluene-1-β-D-deoxyriboside (B) strongly blocks replication (88%) and is mutagenic, causing T>A (24%) mutation. 2,4-Diiodo-5-toluene-1-β-D-deoxyriboside (I) strongly blocks replication (90%) and is very mutagenic causing T>A (46%), T>C (1%), and T>G (1%) point mutations and −1 frame shift mutation (6%) [74]. xG is an ‘expanded base’ of dG (retaining the hydrogen-bonding face), which strongly blocks replication (89%) and is very mutagenic, causing G>A (95%) mutation [75]. xA (expanded A) weakly blocks replication (20%) and is not mutagenic; xT (expanded T) weakly blocks replication (27%), but is very mutagenic, causing T>A (73%) mutation; xC (expanded dC) strongly blocks replication (71%) and is mutagenic, causing C>A (10%) mutation [75].

The α-anomer of deoxynucleosides (α-dN) can be generated as a result of hydroxyl radical attack on deoxyribose [76]. All α-dNs except α-dA strongly block replication in *E. coli* [76]. α-dC blocks almost 99% replication and causes 72% C>A mutation [76]. α-dG also strongly blocks replication and causes 60% G>A mutation [76]. α-dT blocks almost 99% replication but it is not mutagenic in WT *E. coli* [76]. α-dA is not mutagenic [76]. The anticancer agent 6-thioguanine (sG) and its derivative *S*^6^-methylthioguanine (*S*^6^mG) do not block replication strongly in both *E. coli* and human cells [78]. sG causes 11% G>A mutation and *S*^6^mG causes 94% G>A mutation in *E. coli* [78]. sG is less mutagenic (8%) than *S*^6^mG (40%) in human cells as well [78]. Guanine-*S*^6^-sulfonic acid (SO_3_HG) is another derivative of sG [78]. It is not a strong replication block in *E. coli*, but it is very mutagenic, causing 77% G>A mutation [78]. The anti-HIV drug KP1212 is an analog of deoxycytidine [57]. It does not block replication in *E. coli*, but is mutagenic causing 10% C>T mutation [57]. Among the four 2′-deoxyxylonucleosides (xN), only xA and xG exhibit a replication block in *E. coli* [77]. xA is the only mutagenic lesion among the four and causes 10% A>G mutation [77]. Base J strongly blocks replication by 48%, but is not mutagenic in human cells [60].

## 3. Perspectives

In this review, we survey the biological effects of various DNA lesions or biomarkers studied by the shuttle vector techniques, allowing one to gain insight into how DNA damage or other chemically defined nucleobases are processed by polymerases and repair machinery in a natural cellular environment under physiological conditions. Among the new methods that have been developed or applied in the last decade, MS-based strategies and NGS methods have been demonstrated to be efficient for analyzing the lesion’s biological outcomes. LC-MS-based methods are sensitive and accurate for quantifying the degree of lesion bypass and point mutations [4,5]. NGS techniques allow for a large-scale population analysis on many samples at the same time and provide information on a genomic perspective [4,8]. Another possible direction for using vectors as probes to analyze biomarkers is to study the mutational spectrum or mutational signature of a certain chemical or damaging agent [83,84,85,86]. LC-MS- and NGS-based analyses not only consider the biological consequences at the lesion site, but also incorporate information from the neighboring bases, such as one or two nucleotides next to the lesion site from both the 5′ and 3′ direction. An oligonucleotide containing the modified base can be made surrounded by nearest (and next-to nearest) randomized bases and ligated into a shuttle vector. While cellular analysis may pull out a hotspot consensus sequence for poor repair and/or mutagenic replication, this will not answer the primary question of contextual bias in adduct formation. Shuttle vector systems whereby the vector is treated with the chemical to be assessed, followed by quantification of adduct type and amount, and transfection into isogenic cells of varying repair and/or replication backgrounds may tease apart the contribution of local sequence environment to adduct formation, repair, and replication. Such vectors were used over a decade ago [87], and coupled with NGS throughput and bioinformatics, may provide enough reads to make statistically significant claims. Shuttle vectors are currently, to our knowledge, mainly DNA-based; however, one can envision use of RNA-based vectors to study the effect of modified RNA bases on cellular processes such as viral replication, translation, reverse transcription, and possibly even repair. While the role of DNA damage in toxicology focuses mainly on the direct adduction of chemical damage to DNA, pool mutagenesis has often been overlooked, and it would be interesting to leverage shuttle vector techniques to study the incorporation of modified bases from the nucleotide pool in the form of damaged DNA or from DNA-based therapeutics.

## Figures and Tables

**Table 1:** Bypass efficiency and mutagenicity of DNA modifications.

**Oxidative Lesion**	**Bypass Efficiency**	**Mutation**	**Cell**
8-oxo-G	88% [28,29]	G>T 3% (44%, MutY-) [28,29]	*E. coli*
		G>T 8% [30]	Human
Fapy-dG	31–43% (TXN sequences) [31]	G>T 1.2–1.9% (0.7–2.1%, MutM-/MutY-) [31]	*E. coli*
		G>T 10% [30]	Human
NI	7% (57%, SOS) [32]	G>C 8.9%, G>A 19%, G>T 22% (G>C 2.5%, G>A 13%, G>T, 18%, SOS) [32]	*E. coli*
Oa	52% [28], 51% [33], 108% (118% MutY-) [29]	G>T 97% [28], 99% [33], 97% (no change, MutY-) [29]	*E. coli*
Oz	57% [28]	G>T 86% [28]	*E. coli*
Ca	65% [28]	G>T 95% [28]	*E. coli*
Gh	75% [34], 20% (30% MutY-) [29]	G>C 98%, G>T 2% [34], G>T 40%, G>C 57%, G>A 3% (no change, MutY-) [29]	*E. coli*
Sp1	9% [34], 19% (38%, MutY-) [29]	G>C 72%, G>T 27% [34], G>T 78%, G>C 19%, G>A 1% (no change, MutY-) [29]	*E. coli*
Sp2	9% [34], 17% (30%, MutY-) [29]	G>C 57%, G>T 41% [34], G>T 49%, G>C 48%, G>A 3% (no change, MutY-) [29]	*E. coli*
Ur	11% [35], 10% (10% MutY-) [29]	G>T 99% [35], G>T 54%, G>C 35%, G>A 9% (no change, MutY-) [29]	*E. coli*
Iz	60% (71%, SOS) [32]	G>C 88%, G>A 2%, G>T 1.1% (G>C 75%, G>A 3.4%, G>T 5.5%, SOS) [32]	*E. coli*
Cyclo-dG	11% (6% pol V-) [36]	G>A 20% [36]	*E.coli*
S-cdG	4% [37]	G>T 35%, G>A 20% [37]	Human
Cyclo-dA	31% (13% pol V-) [36]	A>T 11% [36]	*E. coli*
S-cdA	6% [37]	A>T 12% [37]	Human
Tg	96% [38]		*E. coli*
5ClC	75% (75% AlkB-) [39]	C>T 5% (same in AlkB-) [39]	*E. coli*
5-OH-C		C>T 0.05%, C>G 0.001% [40]	*E. coli*
5-OH-U		C>T 83% [40]	*E. coli*
Ug		C>T 80% [40]	*E. coli*
THF AP site	6% [28], 5.8% [32], 4% (4% MutY-) [29]	AP>T 50%, AP>C 26%, AP>A 7%, -1 del 13% (no change, MutY-) [29]	*E. coli*
2-deoxyribonolactone	5%, (3% pol II-), (1% pol V-) [41]	T 35%, C 42%, A 12%, G 8%, 5′ T (T 42%, C 38%, A 6%, G 14%, 5′ C) [41]	*E. coli*
**Alkyl Modification**	**Bypass Efficiency**	**Mutation**	**Cell**
m1G	15% (3%, AlkB-) [9], 20% (2%, AlkB-) [33]	G>T 3% (G>T 57%, G>A 17%, G>C 6%, AlkB-) [9], G>T 4%, G>A 2% (G>T 52%, G>A 20%, G>C 4%, AlkB-) [33]	*E. coli*
m2G	90% (84% AlkB-; 98% DinB-; 96% AlkB- and DinB-) [42]	G>A 3% (2.7%, AlkB-; 3%, DinB-; 3%, AlkB- and DinB-) [42]	*E. coli*
e2G	100% (98% AlkB-), (106% DinB-), (99% AlkB- and DinB-) [42]	G>A 2%, G>C 1% (G>A 2.3%, AlkB-), (G>A 2%, AlkB- and DinB-) [42]	*E. coli*
*N*^2^-CMdG	100% [43]	Not mutagenic [43]	Mouse
R-*N*^2^-CEdG	39% (13% pol V-) [44]	Not mutagenic [44]	*E. coli*
	100% [43]	Not mutagenic (G>A 23%, G>T 15%, pol κ-) [43]	Mouse
S-*N*^2^-CEdG	75% (28% pol V-) [44]	Not mutagenic [44]	*E. coli*
	99% [43]	Not mutagenic (G>A 23%, G>T 15%, pol κ-) [43]	Mouse
FF	101% (100% AlkB-), (28% DinB-), (36% AlkB- and DinB-) [42]	G>C 1%, (G>A 1%, G>T 1%, AlkB-DinB-) [42]	*E. coli*
HF	92% (88% AlkB-), (28% DinB-), (40% AlkB- and DinB-) [42]	G>C 2% [42]	*E. coli*
*O*^6^mG		G>A 99% [45,46]	*E. coli*
*O*^6^-POB-dG	70% [47]	G>A 90%, G>T 2.5% [47]	*E. coli*
*O*^6^-PHB-dG	40% [47]	G>A 95% [47]	*E. coli*
*O*^6^-CM-dG	10% [47]	G>A 10% [47]	*E. coli*
	40% [48]	G>A 6% [48]	Human
*O*^6^-ACM-dG	2% [47]	G>A 30% [47]	*E. coli*
*O*^6^-HOEt-dG	15% [47]	G>A 40% [47]	*E. coli*
PdG	25% [12]	G>T 6% [12]	Human
α-OH-PdG	17% [12]	G>T 11% [12]	Human
γ-OH-PdG	73% [12]	Not mutagenic [12]	Human
1,*N*^2^-eG	4% (2% AlkB-) (1.8% AlkB-DinB-) [8]	G>A 6%, G>T 6%, G>C 2%, −1/2 del 5% (G>A 13%, G>T 13%, G>C 1%, −1/2 del 9%, AlkB-), (same in AlkB-DinB-) [8]	*E. coli*
2′-F-*N*^2^,3-eG	21% (26% AlkB-) (14% AlkB-DinB-) [8]	G>A 30% (30% AlkB-), (30% AlkB-DinB-) [8]	*E. coli*
m1A	100% (12%, AlkB-) [9]	A>T 0.06% (0.61%, AlkB-) [9]	*E. coli*
eA	85% (5% AlkB-) [33], 130% (9% AlkB-) [49]	<0.5% (A>T 25%, A>G 5%, A>C 5%, AlkB-) [33], A>C 1%, A>T 1% (A>T 22%, A>C 8%, A>G 7%, AlkB-) [49]	*E. coli*
	17% [50]		Human
EA	135% (14% AlkB-) [49]	A>C 1%, A>G 0.5%, A>T 0.5% (A>C 2%, A>G 1%, A>T 1%, AlkB-) [49]	*E. coli*
*N*^6^-CMdA	98% [36]	Not mutagenic [36]	*E. coli*
	65% (35% pol k-) [48]	Not mutagenic [48]	Human
*S*-*N*^6^-HB-dA	120% [51]	Not mutagenic [51]	*E. coli*
*R*,*R*-*N*^6^,*N*^6^-DHB-dA	100% [51]	<1% [51]	*E. coli*
*S*,*S*-*N*^6^,*N*^6^-DHB-dA	60% [51]	A>G 1% [51]	*E. coli*
*R*,*S*-1,*N*^6^-*γ*-HMHP-dA	10% [51]	A>T 2% [51]	*E. coli*
*O*^2^-MedT	60% [52], 55% [53]	T>A 1%, T>G 1% [52], T>A 56% [53]	Human
	5% [54]	T>A 10%, T>G 10% [54]	*E. coli*
*O*^2^-EtdT	21% (5% pol V-) [55]	T>C 35%, T>A 15%, T>G 5% (T>C 10%, pol V-) [55]	*E. coli*
	45% [52]	T>A 5%, T>G 3% [52]	Human
*O*^2^-*n*PrdT	35% [52]	T>A 12%, T>G 5% [52]	Human
*O*^2^-*i*PrdT	35% [52]	T>A 4%, T>G 1% [52]	Human
*O*^2^-*n*BudT	30% [52]	T>A 13%, T>G 6% [52]	Human
*O*^2^-*i*BudT	15% [52]	T>A 4%, T>G 2% [52]	Human
*O*^2^-*s*BudT	15% [52]	T>A 4%, T>G 2% [52]	Human
*O*^2^-POB-dT	3% [54]	12% T>A, 38% T>G [54]	*E. coli*
	26% [53]	T>A 47% [53]	Human
m3T	6%, (4% AlkB-) [9]	T>A 32%, T>C 6%, T>G 2% (T>A 47%, T>C 9%, T>G 2%, AlkB-) [9]	*E. coli*
N3-EtdT	17% (3% pol V-) [55]	T>C 15%, T>A 21%, T>G 3% (Not mutagenic, pol V-) [55]	*E. coli*
N3-CMdT	55% [36]	T>A 66% [36]	*E. coli*
	40% [48]	T>A 81% [48]	Human
*O*^4^-CMdT	49% [36]	T>C 86% [36]	*E. coli*
	40% [48]	T>C 68% (25% pol ζ-) [48]	Human
*O*^4^-MedT	32% [56]	T>C 58% [56]	Human
*O*^4^-EtdT	76% [55]	T>C 84% (Not mutagenic, pol V-) [55]	*E. coli*
	33% [56]	T>C 82% [56]	Human
*O*^4^-*n*PrdT	35% [56]	T>C 42% [56]	Human
*O*^4^-*i*PrdT	30% [56]	T>C 44% [56]	Human
*O*^4^-*n*BudT	32% [56]	T>C 29% [56]	Human
*O*^4^-*i*BudT	24% [56]	T>C 42% [56]	Human
*O*^4^-R-*s*BudT	20% [56]	T>C 25% [56]	Human
*O*^4^-S-*s*BudT	22% [56]	T>C 25% [56]	Human
m3C	100% (10% AlkB-) [9], 113% (14% AlkB-) [57], 98% (5% AlkB-; 115% DinB-; 7.5% AlkB-DinB-) [42], 100% (15% AlkB-) [39]	C>T 1% (C>T 14%, C>A 14%, C>G 2%, AlkB-) [9], Not mutagenic (C>T 55%, C>A 30%, C>G 1%, AlkB-) [57], Not mutagenic (C>T 41%, C>A 41%, C>G 4%, AlkB-) [42], Not mutagenic (C>T 52%, C>A 30%, AlkB-) [39]	*E. coli*
e3C	96%, (9% AlkB-) [9]	Not mutagenic (C>T 17%, C>A 11%, C>G 2%, AlkB-) [9]	*E. coli*
*N*^4^-CMdC	83% [36]	Not mutagenic [36]	*E. coli*
	80% [48]	Not mutagenic [48]	Human
5mC	100% (100% AlkB-) [39]	Not mutagenic (same in AlkB-) [39]	*E. coli*
	100% [58]	Not mutagenic [58]	Human
5hmC	100% [59]	Not mutagenic [59]	*E. coli*
	98% [58]	Not mutagenic [58]	Human
5fC	100% [59]	Not mutagenic [59]	*E. coli*
	74% [58]	Not mutagenic [58]	Human
5caC	100% [59]	Not mutagenic [59]	*E. coli*
	72% [58]	Not mutagenic [58]	Human
eC	24% (13% AlkB-) [33]	C>A 24%, C>T 11% (C>A 49%, C>T 31%, AlkB-) [33]	*E. coli*
H-edC	1% [50]	C>G 40% [50]	*E. coli*
	10% [50]	C>A 60%, C>T 32% [50]	Human
5hmU	80% [60]	Not mutagenic [60]	Human
Sp-Me-PTE	110% (Ada-, decreases from 140% to 70%) [61]	TT>GT 50%, TT>GC 15% [61]	*E. coli*
Rp-Me-PTE	30% [61]	Not mutagenic [61]	*E. coli*
Sp-Et-PTE	190% [61]	Not mutagenic [61]	*E. coli*
Rp-Et-PTE	40% [61]	Not mutagenic [61]	*E. coli*
Sp-*n*Pr-PTE	160% [61]	Not mutagenic [61]	*E. coli*
Rp-*n*Pr-PTE	70% [61]	Not mutagenic [61]	*E. coli*
Sp-*n*Bu-PTE	100% [61]	Not mutagenic [61]	*E. coli*
**Bulky Lesion**	**Bypass Efficiency**	**Mutation**	**Cell**
*N*^2^-MC-dG	38% [62]	G>T 18% [62]	Human
*N*^2^-2,7-DAM-dG	27% [62]	G>T 10% [62]	Human
AL-II-dG	9% [63]	G>T 9% [63]	Mouse
AFB_1_-N7-dG		G>T 1.5% [64]	*E. coli*
AFB_1_-FAPY		G>T 14% [65]	*E. coli*
C8-AP-dG	51% [66]	Not mutagenic [66]	Human
C8-AAF-dG	13% [66]	Not mutagenic [66]	Human
C8-AF-dG	97% [66]	Not mutagenic [66]	Human
AL-I-dA	100% (5% Rev3L-) [67]	A>T 50% (Not mutagenic, Rev3L-) [67]	Mouse
AL-II-dA	5% [63]	A>T 22% [63]	Mouse
BPDE-dG	(40% Rev1-); (13% Rev3L-) [68]	G>T 73%, G>A 12%; (G>T 32%, G>A 18%, Rev1-); (G>T 6%, Rev3L-) [68]	Mouse
**Crosslinked Lesion**	**Bypass Efficiency**	**Mutation**	**Cell**
ICL-RD	43% [69]	5′-G>T 3% [69]	*E. coli*
ICL-R	38% [69]	5′-G>T 3% [69]	*E. coli*
ICL-S	53% [69]	5′-G>T 3% [69]	*E. coli*
AP-dG (dG strand)	38% (43% Pol η-), (13% Pol ι-), (2% Pol κ-), (5% Pol ζ-) [70]	G>A 2-5%, G>T 1–2%, G>C 1% [70]	Human
AP-dG (AP strand)	18% (25% Pol η-), (4% Pol ι-), (1% Pol κ-), (5% Pol ζ-) [70]	AP>T 74%, AP>C 10-20%, AP>G 4–6%, AP>A 1–2% [70]	Human
1,2-GG-*cis*-DDP	11% [71]; 5% (30% SOS) [72]	<0.25% (G>T 1.3%, SOS) [72]	*E. coli*
1,2-AG-*cis*-DDP	22% (32% SOS) [72]	<0.2% (A>T 4.4%, SOS) [72]	*E. coli*
1,3-GTG-*cis*-DDP	13% (14% SOS) [72]	<0.7% [72]	*E. coli*
γ-HOPdG mediated peptide crosslink		G>T 5%, G>C 3% [20]	Human
γ-HOPdA mediated peptide crosslink		Not mutagenic [20]	Human
5fC mediated peptide crosslink		C>T 7%, C>G 1%, C del 2% [73]	Human
**Other Nucleotide Analog**	**Bypass Efficiency**	**Mutation**	**Cell**
H	5% [74]	T>A 41%, T>C 5%, T>G 4%, −1 del 13% [74]	*E. coli*
F	13% [74]	T>A 9%, T>C 1%, T>G 1% [74]	*E. coli*
L	20% [74]	T>A 5% [74]	*E. coli*
B	12% [74]	T>A 24% [74]	*E. coli*
I	10% [74]	T>A 46%, T>C 1%, T>G 1%, −1 del 6% [74]	*E. coli*
KP1212	128% [57]	C>T 10% [57]	*E. coli*
xG	11% (45% SOS) [75]	G>A 95% [75]	*E. coli*
xA	80% (108% SOS) [75]	<1% [75]	*E. coli*
xT	73% (102% SOS) [75]	T>A 73% [75]	*E. coli*
xC	29% (53% SOS) [75]	C>A 10% [75]	*E. coli*
α-dG	3% [76]	G>A 60%, G>C 6% [76]	*E. coli*
α-dA	20% [76]	Not mutagenic [76]	*E. coli*
α-dT	1% [76]	Not mutagenic [76]	*E. coli*
α-dC	1% [76]	C>A 72% [76]	*E. coli*
dxG	25% [77]	Not mutagenic [77]	*E. coli*
dxA	75% [77]	A>G 10% [77]	*E. coli*
dxT	150% [77]	Not mutagenic [77]	*E. coli*
dxC	125% (CXT), 175%(GXG) [77]	Not mutagenic [77]	*E. coli*
sG	98% [78]	G>A 11% [78]	*E. coli*
	98% [79]	G>A 8% [79]	Human
*S*^6^mG	91% [78]	G>A 94% [78]	*E. coli*
	95% [79]	G>A 40% [79]	Human
SO_3_HG	87% [78]	G>A 77% [78]	*E. coli*
2′-F-G	99% [8]	Not mutagenic [8]	*E. coli*
J	52% [60]	Not mutagenic [60]	Human
